# Intra-abdominal hypertension in patients with severe acute pancreatitis

**DOI:** 10.1186/cc3754

**Published:** 2005-07-06

**Authors:** Jan J De Waele, Eric Hoste, Stijn I Blot, Johan Decruyenaere, Francis Colardyn

**Affiliations:** 1 Intensivist, Intensive care unit, Ghent University Hospital, Gent, Belgium; 2 Professor, Intensive care unit, Ghent University Hospital, Gent, Belgium; 3 Professor and Head, Intensive care unit, Ghent University Hospital, Gent, Belgium; 4 Professor and Chief Executive Officer, Ghent University Hospital, Gent, Belgium

## Abstract

**Introduction:**

Abdominal compartment syndrome has been described in patients with severe acute pancreatitis, but its clinical impact remains unclear. We therefore studied patient factors associated with the development of intra-abdominal hypertension (IAH), the incidence of organ failure associated with IAH, and the effect on outcome in patients with severe acute pancreatitis (SAP).

**Methods:**

We studied all patients admitted to the intensive care unit (ICU) because of SAP in a 4 year period. The incidence of IAH (defined as intra-abdominal pressure ≥ 15 mmHg) was recorded. The occurrence of organ dysfunction during ICU stay was recorded, as was the length of stay in the ICU and outcome.

**Results:**

The analysis included 44 patients, and IAP measurements were obtained from 27 patients. IAH was found in 21 patients (78%). The maximum IAP in these patients averaged 27 mmHg. APACHE II and Ranson scores on admission were higher in patients who developed IAH. The incidence of organ dysfunction was high in patients with IAH: respiratory failure 95%, cardiovascular failure 91%, and renal failure 86%. Mortality in the patients with IAH was not significantly higher compared to patients without IAH (38% versus 16%, p = 0.63), but patients with IAH stayed significantly longer in the ICU and in the hospital. Four patients underwent abdominal decompression because of abdominal compartment syndrome, three of whom died in the early postoperative course.

**Conclusion:**

IAH is a frequent finding in patients admitted to the ICU because of SAP, and is associated with a high occurrence rate of organ dysfunction. Mortality is high in patients with IAH, and because the direct causal relationship between IAH and organ dysfunction is not proven in patients with SAP, surgical decompression should not routinely be performed.

## Introduction

Despite recent advances in the management of patients, such as early enteral nutrition and withholding surgery until proven infection of pancreatic necrosis, severe acute pancreatitis (SAP) remains a disease with an unpredictable clinical course and significant morbidity and mortality [1]. Infection still remains the most feared complication, but also the presence of organ dysfunction is increasingly recognized as an important risk factor for mortality in patients with severe disease [2-4].

Intra-abdominal hypertension (IAH) has been recognized as a cause of organ dysfunction in critically ill patients, with respiratory and renal dysfunction often most prominent [5]. This syndrome, referred to as the abdominal compartment syndrome, has most extensively been described in patients who underwent emergency abdominal surgery or after abdominal trauma, but also in patients with non-abdominal diseases such as burns [6] and massive fluid resuscitation [7].

Some recent studies [8,9] suggest that IAH is a frequent finding in SAP patients. The clinical relevance of this remains unclear, although Pupelis *et al*. [9] found a relation between elevated intra-abdominal pressure (IAP; above 25 mmHg) and persistent subsequent organ dysfunction. Tao *et al*. [10] described a high incidence of IAH in patients with early SAP, but lack of a definition of IAH and methodological issues make interpretation of these data difficult [10].

The levels at which elevated IAP can cause organ dysfunction are lower than in the study by Pupelis *et al*. Values of as low as 15 mmHg may result in clinically significant organ damage [11-13], but clinical significance of this lower threshold in patients with SAP remains to be determined.

The aim of this analysis was to study patient factors associated with the development of IAH. Furthermore, we studied the incidence of organ failure in patients with SAP and IAH, and the association of the presence of IAH and outcome.

## Materials and methods

### Patients

We studied all patients admitted because of SAP to the intensive care unit (ICU) of the Ghent University Hospital (Gent, Belgium) between January 2000 and March 2004. SAP was defined according to the criteria described by the International Symposium on Acute Pancreatitis [14]. Patients names were retrieved from the hospital registry using ICD code 577.0 (acute pancreatitis), and files were reviewed retrospectively. Patients who were referred from other hospitals later than 7 days after the start of SAP were excluded. The study was approved by the local ethical committee.

Preoperative data collected included age, gender, etiology of SAP, C-reactive protein level, Ranson score and Acute Physiology And Chronic Health Evaluation (APACHE) II score [15] on admission and C-reactive protein at 48 h after admission.

### Data acquisition

IAP values were measured every 8 h when IAP was below 15 mmHg, and every 4 h when above 15 mmHg, and were retrieved from the patients file. IAP was measured using the transvesical route, as described by Cheatham *et al*. [16], after instillation of 50 ml of saline in the bladder. IAP measurements were obtained from patients when multiple intra-abdominal fluid collections were present on CT scan on admission, or when there was the clinical suspicion of IAH. These clinical indications included oliguria, hypoxia, abdominal distension, and severe abdominal pain. The incidence of IAH (defined as IAP ≥ 15 mmHg) was recorded, as was the maximal IAP value obtained during ICU stay, and the duration of IAP levels ≥ 15 mmHg.

The occurrence rate of organ dysfunction during ICU stay was recorded and defined as: cardiovascular, hypotension requiring vasoactive medication (epinephrine, norepinephrine, dobutamine at any dose, or dopamine at doses above 2 mcg/kg/min); renal, serum creatinine above 2.0 mg/dl; pulmonary, the need for mechanical ventilation or PaO2/FiO2 ratio < 300. Mortality was defined as in-hospital mortality.

Interventions to alleviate IAH were recorded, as were complications of these interventions. Decompressive laparotomy was considered when rapidly deteriorating, therapy resistant multiple organ dysfunction was present in the first days after admission, and decided on a patient to patient basis.

### Statistical analysis

Statistical analysis was performed using SPSS for Windows 11.0.1 ^® ^(SPSS, Chicago, IL, USA). Continuous variables were compared using the Mann Whitney U-test. Categorical data were compared using the Chi-square or Fisher Exact test. Continuous data are expressed as mean (standard deviation) if the data were normally distributed, or median (interquartile range) if the distribution was not normal. Categorical data are reported as n (%). Pearson correlation coefficient between maximal IAP and APACHE II score was calculated. Mean IAP values from day 1 to 7 were compared using the Friedman test. A double sided p-value of less than 0.05 was considered statistically significant.

## Results

### General

Forty-four patients were admitted to the ICU because of SAP during the study period. Mean age was 57 years (15.8) and 27 were male (61%). The etiology of acute pancreatitis was biliary tract stones in 19 patients, alcohol intake in 12, hyperlipemia in 4, and trauma in 2. In 7 patients, the cause of pancreatitis could not be determined. Mean Ranson score of the patients was 5.5 (2.6), mean APACHE II score was 18 (9.2).

### IAP monitoring

**Figure 1:**
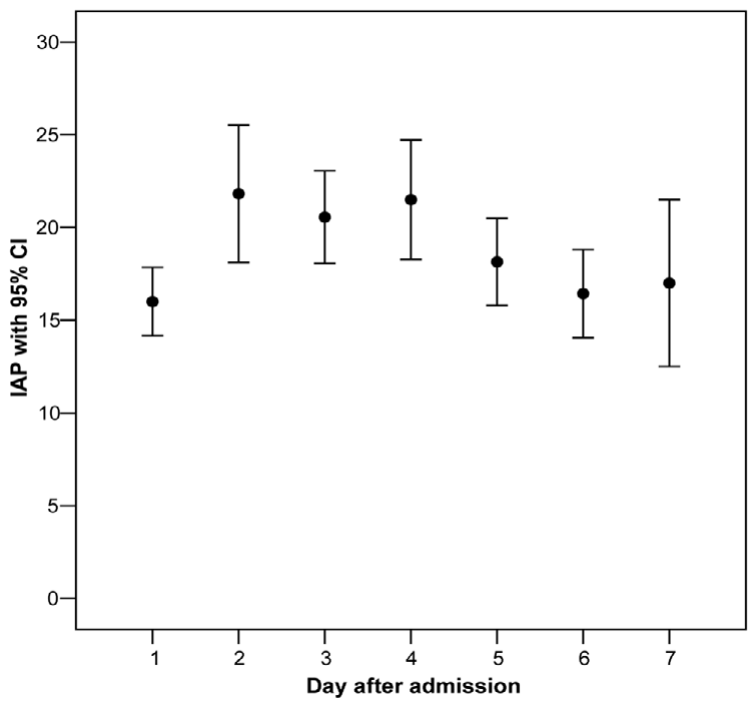
Evolution of intra-abdominal pressure (IAP) in the first week after admission. Mean IAP with 95% confidence interval (CI).

IAP measurements were obtained from 27 patients, but in the remaining 17 patients IAP was not measured. Of the 27 patients, 21 developed IAH (78%). In 12 patients, IAP monitoring was available from the first day of admission to the ICU. In these 12 patients, IAH developed after a median of 1 day after admission to the hospital and mean IAP increased from 16 at day 1 to 22 mmHg the day after, and remained elevated. There was a trend towards a significant difference between the mean IAP values during the first week of admission (p = 0.12) (Fig. 1).

**Figure 2:**
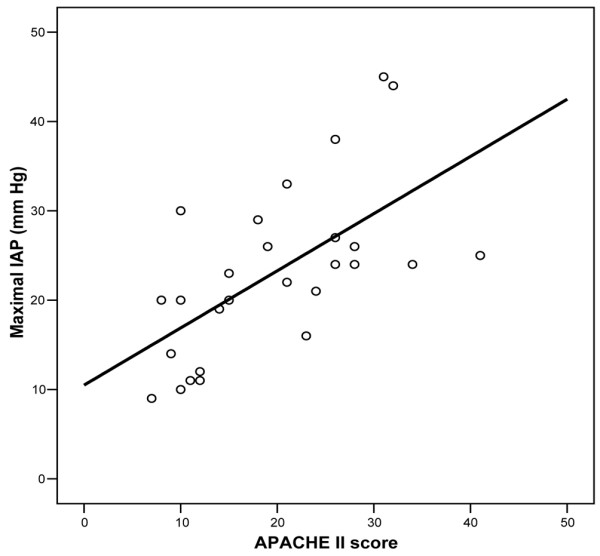
Correlation between maximal intra-abdominal pressure and APACHE II score in patients with severe acute pancreatitis.

The maximum IAP in patients with IAH averaged 27 (7.8) mmHg. In patients who did not undergo abdominal decompression (n = 17), IAH persisted for a median of 6 days (interquartile range 3–8). Maximal IAP correlated significantly with APACHE II score (correlation coefficient 0.60, p < 0.002) (Fig. 2).

### Factors associated with IAH

In univariate analysis, the APACHE II and Ranson scores on admission were higher in patients who developed IAH (Table 1). Age, gender, cause of pancreatitis and C-reactive protein levels at 48 h were not significantly different between the two groups. Pancreatic necrosis was documented in all but one patient who developed IAH, whereas only three patients without IAH had pancreatic necrosis; the other three patients had pancreatic oedema and peripancreatic fluid collections on CT scan.

### Organ dysfunction, surgical interventions and outcome

The incidence of organ dysfunction was higher in patients with IAH compared to patients without IAH (Table 1). Thirteen patients with IAH were treated with renal replacement therapy compared to none in the patients without IAH. Duration of mechanical ventilation was maintained for 15 (12.6) days in patients with IAH.

**Figure 3:**
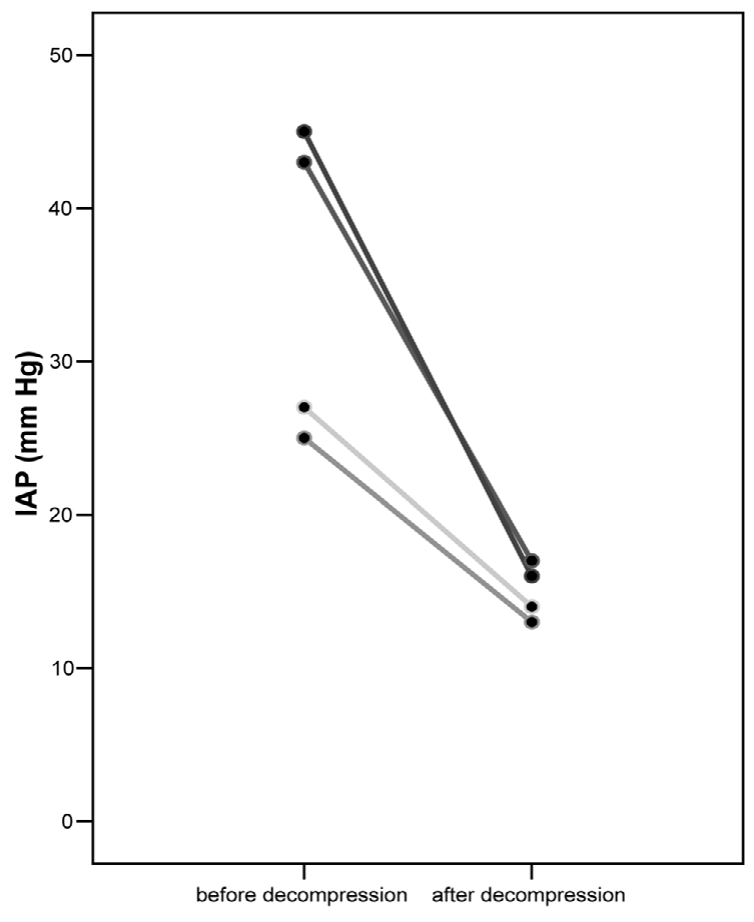
Effect of surgical decompression on intraabdominal pressure.

Surgical treatment was more frequent in patients with IAH. Of the 21 patients with IAH, 9 were treated surgically, whereas no patient in the non-IAH group needed surgery (p = 0.07). The indication for surgery was abdominal compartment syndrome in four patients and infected pancreatic necrosis in five patients. Abdominal decompression was performed surgically through a midline laparotomy. In four patients, a temporary abdominal closure system was used because of abdominal compartment syndrome with IAP ranging from 25 to 45 mmHg. IAP decreased in all patients (Fig. 3). In one patient, necrosectomy was performed at the time of decompression. Three of these patients died early in the postoperative course. The cause of death was uncontrollable retroperitoneal bleeding in two patients, and further deterioration of organ dysfunction in another patient. Patients with IAH stayed significantly longer in the ICU and in the hospital than patients without IAH (Table 1).

Mortality in the patients with IAH was not significantly higher than in patients without IAH (8/21 (38%) versus 1/6 (16%), p = 0.63). The non-IAH patient died after therapy was withdrawn early after ICU admission because of a concomitant advanced brain tumour. Four patients with IAH died within 4 days after hospital admission, three of them within 24 h after surgical decompression. The four other patients with IAH with fatal outcomes died on day 12, 26, 35 and 38 because of persistent organ dysfunction, in association with infected pancreatic necrosis in three patients.

## Discussion

In this cohort of patients admitted to the ICU because of SAP, the incidence of IAH was 51%. When only patients in who IAP monitoring was performed are considered, the incidence reached 78%, but this might be an overestimation as IAP measurement was not performed routinely and was based upon clinical suspicion for IAH. Also, IAH developed early in the course of the disease; in the majority of the patients in whom IAP monitoring was available from the day of admission, IAH developed within 24 h after ICU admission.

Although the difference in IAP during the first week was not significant, there seem to be three time frames early in the course of the disease. At day 1 the IAP was already elevated, and it then increased to the maximal level at day 2 and remained elevated until day four after admission. IAH in patients with SAP seems to be an early event.

Maximal IAP values were well above the 15 mmHg threshold used for the definition of IAH, and were as high as 25 to 40 mmHg in some patients, including the four patients who underwent abdominal decompression for abdominal compartment syndrome. These high values of IAP may be an explanation for the high incidence of organ failure in these patients, as all patients with IAH developed at least one organ failure, and the majority two or more.

SAP patients develop IAH for several reasons. Pancreatic or retroperitoneal inflammation is the most obvious reason in the early course of the disease. Aggressive fluid resuscitation, resulting in generalized and visceral odema in particular, will add to the intra-abdominal volume during the first days of severe disease. Furthermore, paralytic ileus and peripancreatic acute fluid collections can also increase IAP.

From the APACHE II and Ranson scores of the patients, it seems that the more severe the disease, the higher the likelihood to develop IAH. But IAH itself may be an early predictor of severe disease, as elevated IAP seems to occur early in the course of the disease. IAH may even contribute to disease severity in patients with SAP, but the exact role remains to be determined. Elevated IAP causes intestinal hypoperfusion even at levels as low as 8 to 12 mmHg [12]. In the setting of SAP, pancreatic perfusion may also be affected, and possibly IAH may contribute to the development of pancreatic hypoperfusion and eventually pancreatic necrosis. The observation of increased bacterial translocation in patients with IAH and abdominal compartment syndrome [17] may also apply to patients with SAP. Animal studies have shown an increased rate of bacterial translocation in acute pancreatitis [18], but the role of IAH in this remains to be elucidated.

Patients with IAH had necrosis more often and were operated on more often. This resulted in a longer ICU and hospital stay for these patients. Surgical decompression was performed in four patients with IAP levels above 25 mmHg and severe organ dysfunction, but only one patient survived. The three other patients succumbed early after decompression, two patients from hemorrhagic shock and one from further deteriorating multiple organ dysfunction syndrome. The necrosectomy that was performed in the first patient treated with abdominal decompression possibly played a role in the hemorrhagic shock and deterioration early after surgery. Necrosectomy was not applied to subsequent patients who underwent decompression. In the second patient who died of uncontrollable bleeding from the retroperitoneum, the bleeding itself may have played a role in the development of IAH. At laparotomy, there was a large retroperitoneal haematoma, with active bleeding, possibly caused by an eroded vessel or pseudoaneurysm. Due to the profound bleeding, no clear cause could be identified and, unfortunately, the family of the patient refused a post mortem examination.

This experience in our four patients has tempered our initial enthusiasm for decompression in patients with IAH and SAP [19]. Other authors also reported poor survival rates after surgical decompression in patients with SAP [8]. Patient selection may, however, bias the results of decompression, as only patients with uncontrollable organ dysfunction have been considered candidates for decompression in our unit, and also the timing of surgical decompression may play a crucial role.

There has been a recent trend towards postponing surgery in patients with SAP because early surgical intervention was associated with an increased mortality rate [20,21]. This could also be concluded from our limited number of patients who died shortly after surgery, but it should be considered that the strategy of early intervention in SAP without infection, where the retroperitoneum is debrided, differs substantially from a procedure in which the abdomen is opened, but the retroperitoneum is left untouched. Moreover, in one of the patients that was decompressed and debrided in our study, an uncontrollable haemorrhage from the retroperitoneum occurred, and the patient died a few hours later.

Little can be concluded from this study as to the usefulness of early debridement but, in our experience, the absence of infection, increased age and acute renal failure were associated with an increased mortality in a series of patients who were treated surgically for severe acute pancreatitis; the timing of the surgical intervention itself had no effect on this [22].

## Conclusion

Severity of disease predisposes for IAH in patients with SAP. The occurrence rate of IAH is high, and IAH is associated with organ dysfunction in the majority of patients. Mortality is high in patients with IAH, but it is not clear if surgical decompression in these patients is advantageous.

## Key messages

• 	IAH is a frequent finding in critically ill patients with SAP

• 	The maximum IAP is related to the severity of illness

• 	Organ dysfunction is present in patients with moderately increased IAPs

• 	Abdominal decompression was associated with a 75% mortality in this study

## Abbreviations

APACHE = Acute Physiology And Chronic Health Evaluation; IAH = intra-abdominal hypertension; IAP = intra-abdominal pressure; ICU = intensive care unit; SAP = severe acute pancreatitis.

## Competing interests

The authors declare that they have no competing interests.

## Authors' contributions

JDW and EH conceived and designed the study. Acquisition of a substantial portion of data was done by JDW. Analysis and interpretation of data was performed by JDW, EH and SB. JDW and SB drafted the manuscript. FC, JDC and EH critically revised the manuscript for important intellectual content. EH and SB supplied statistical expertise. FC supervised and was responsible overall for all aspects of the study.

## Figures and Tables

**Table 1:** Characteristics of patients who did or did not develop intra-abdominal hypertension during ICU stay (n = 27)

Characteristic	IAH (n = 21)	Non-IAH (n = 6)	p-value
Age	53 (45–68)	46 (26–76)	0.629
Male gender	15 (71%)	4 (67%)	1.000
APACHE II score	21 (15–28)	10 (8–11)	0.005
Ranson score	7 (6–8)	3 (1–5)	0.014
Etiology of pancreatitis			0.552
Biliary	7 (33%)	4 (67%)	
Alcohol	8 (38%)	2 (33%)	
Hyperlipemia	3 (14%)		
Trauma	1 (5%)		
Unknown	2 (10%)		
CRP level 48 h after admission (mg/dL)	34 (19–40)	34 (26–39)	0.521
Pancreatic necrosis	20 (95%)	3 (50%)	0.025
Surgical management	9 (43%)	0 (0%)	0.070
Infected pancreatic necrosis	5 (24%)	0 (0%)	0.555
Organ dysfunction			
Pulmonary failure	20 (95%)	2 (33%)	0.004
Cardiovascular failure	19 (91%)	1 (17%)	0.001
Renal failure	18 (86%)	1 (17%)	0.004
LOS ICU (days)	21 (10–37)	3 (1–5)	0.003
LOS hospital (days)	42 (20–90)	12 (3–14)	0.015

APACHE, Acute Physiology And Chronic Health Evaluation; CRP, C-reactive protein; IAH, intra-abdominal hypertension; ICU, intensive care unit; LOS, length of stay.
